# Quaternary Cu_2_TSiS_4_ (T = Fe, Mn) Anodes for Li-Ion Batteries

**DOI:** 10.1021/acsaem.4c03366

**Published:** 2025-01-18

**Authors:** Eric Youngsam Kim, Zachary T. Messegee, Zhenzhen Yang, Xiaoyan Tan, Chao Luo

**Affiliations:** † Department of Chemistry and Biochemistry, George Mason University, Fairfax, Virginia 22030, United States; ‡ Chemical Sciences and Engineering Division, Argonne National Laboratory, Lemont, Illinois 60439, United States; § Quantum Science & Engineering Center, George Mason University, Fairfax, Virginia 22030, United States; ∥ Department of Chemical, Environmental and Materials Engineering, University of Miami, Coral Gables, Florida 33146, United States

**Keywords:** Li-ion batteries, quarternary transition metal sulfides, anode materials, high cyclic stability, fast-charging capacity

## Abstract

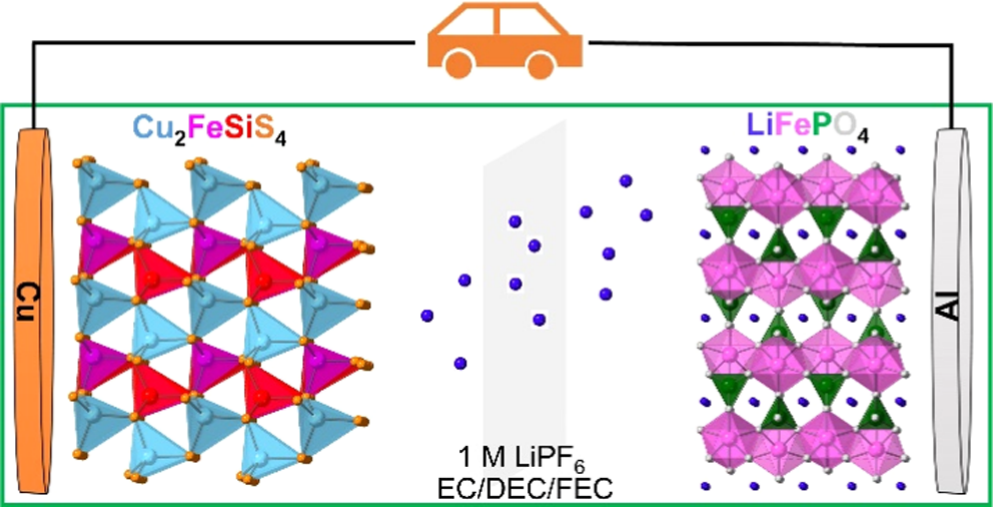

Developing high-capacity and fast-charging anode materials is critical for achieving high-performance Li-ion batteries (LIBs). Herein, polycrystalline quaternary transition metal silicon sulfides, Cu_2_TSiS_4_ (T = Fe, Mn), were synthesized using a solid-state method and investigated as anode materials in LIBs. Cu_2_FeSiS_4_ retains a reversible capacity of 670 mAh g^–1^ at 200 mA g^–1^ for 400 cycles, while Cu_2_MnSiS_4_ suffers from a fast capacity loss in the initial 50 cycles. More importantly, Cu_2_FeSiS_4_ can maintain a reversible capacity of 379 mAh g^–1^ after 700 cycles at a high current density of 2 A g^–1^, demonstrating high cyclic stability and fast-charging capacity. To further understand the structure degradation and phase transformation, we investigated the postcycling electrodes using multiple techniques, including the scanning electron microscope with energy-dispersive X-ray spectroscopy, X-ray diffraction, and X-ray photoelectron spectroscopy techniques. The results indicated that Cu_2_FeSiS_4_ undergoes reversible phase transitions with Li_2_S as a major product component. To further assess the performance for practical applications, Cu_2_FeSiS_4_ was coupled with LiFePO_4_ to make LiFePO_4_||Cu_2_FeSiS_4_ full cells, which delivered superior electrochemical performance. These results demonstrate great promise for using quaternary transition metal silicon sulfides as anodes to achieve low-cost and sustainable LIBs.

## Introduction

Lithium-ion batteries (LIBs) were first commercialized by the Sony company in 1991 and are currently the most widely utilized energy storage devices for consumer electronics, electronic transportation, and grid energy storage due to the high energy density, wide operating voltage window, limited self-discharge, and long lifetime.^1−6^ The large-scale applications of LIBs in various fields have led to demands for energy storage and distribution with high energy density, fast charge/discharge capability, long cycle life, and high safety.^7−9^ However, state-of-the-art commercial graphite-based anodes cannot satisfy these requirements due to the low theoretical capacity of 372 mAh g^–1^ and safety issues caused by the cracks on the electrode surface and the formation of lithium dendrites.^10,11^ Therefore, significant research efforts have been devoted to designing advanced anode materials that offer high capacity, long lifetime, and improved safety.^12^

Anode materials for LIBs can be classified into three categories depending on the reaction mechanisms: intercalation/deintercalation reaction, alloying reaction, and conversion reaction.^13^ Based on these mechanisms, various anode materials such as carbon, silicon, Li metal, transition metal oxides, transition metal chalcogenides (TMCs), transition metal phosphides, and transition metal phosphorus trisulfides have been developed.^14−18^ Among these anode materials, conversion-type TMCs have attracted considerable interest due to their low cost and high specific capacity.^19^ Sulfur has a lower electronegativity value than that of oxygen, resulting in weaker ionic bonding in TMCs than in transition metal oxides. Therefore, TMCs have a smaller band gap energy, higher conversion rates, and higher conductivities.^20−22^ Layered TMCs exhibit high capacity in LIBs because of their stacked arrangement, which tends to accommodate lithium ions in vacancies.^23^ Among the TMCs, transition metal sulfides (TMSs) are highly promising due to their high theoretical capacity (500–1000 mAh g^–1^), relatively low volume expansion, and high thermal stability.^24,25^ For example, layered TiS_2_ has the highest theoretical capacity of 960 mAh g^–1^ among TMSs.^26−28^ However, TMSs suffer from inferior rate capability and structural instability during the repeated lithiation and delithiation processes. For instance, MoS_2_ and CuS_2_ display low-capacity retention after a few cycles due to unstable microsized structures upon cycling.^29−32^ Reducing the particle size of TMSs to the nanoscale enhances the electrochemical performance at the price of higher production cost, lower volumetric capacity, more interphase formation, and increased electrolyte consumption. Consequently, new designs of anode materials, such as binary, ternary, or quaternary TMSs, are necessary to improve the performance. The combination of multiple transition metal elements, silicon, and sulfur to form binary, ternary, or quaternary TMSs offers opportunities to obtain low-cost and high-performance anode materials for LIBs.^33,34^

To this end, TMSs have flexibility in chemical and structural design, providing high tunability of their physical properties. So far, a wide variety of TMSs have been extensively investigated as narrow band gap semiconductors for photovoltaic and thermoelectric materials, enabling efficient charge transport.^35−37^ Recently, a few studies have been extended to develop anode materials for battery applications. For example, Yuan et al. demonstrated the use of Cu_2_NiSnS_4_ nanoparticles integrated with graphene oxide nanosheets in Na-ion batteries.^38^ To date, research on binary, ternary, or quaternary TMSs as anode materials has been focused on developing TMS thin films and nanomaterials, owing to the high performance of nanostructured materials. The study of bulk TMSs as anode materials is demanded but hindered by the poor electrochemical performance due to the poor cycle life of microsized materials.

**Figure 1:**
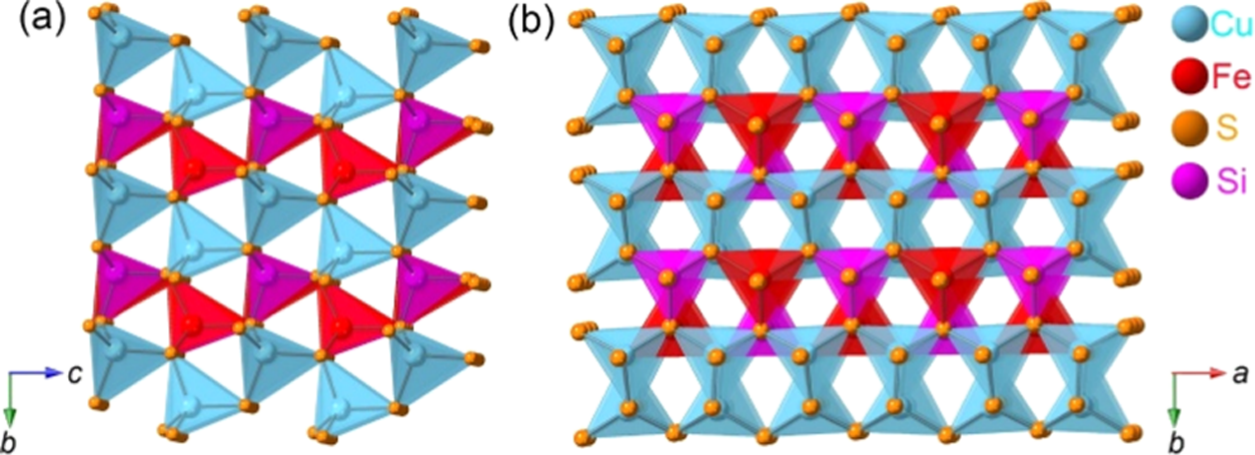
Perspective view of the crystal structure of Cu_2_FeSiS_4_ along the crystallographic *a*-axis (a) and *c*-axis (b).

Herein, we designed and synthesized microsized quaternary TMSs, Cu_2_TSiS_4_ (T = Fe, Mn), using a high-temperature solid-state synthesis. Cu_2_TSiS_4_ (T = Fe, Mn) adopts the orthorhombic wurtz-stannite structure type with the space group *Pmn*2_1_ (Figure 1).^39^ The crystal structure can be described as a superstructure of the wurtzite (ZnS) structure with the hexagonally closest packed S anions and half of the tetrahedral holes occupied by metal cations. The formed tetrahedral coordination in CuS_4_, MnS_4_/FeS_4_, and SiS_4_ tetrahedra is similar to those in the stannite (Cu_2_FeSnS_4_) structure. As shown in Figure 1, the crystal structure of Cu_2_FeSiS_4_ contains zigzag chains of CuS_4_, FeS_4_, and SiS_4_ tetrahedra, which are linked via corner-sharing. The bond distances in each tetrahedron are not equal, which causes distortion in the tetrahedra, and the whole crystal structure is noncentrosymmetric. The metal-rich sites in these materials could improve the conductivity and stability. This work explores the correlation between their structures and electrochemical performance, offering guidance for further structure design and performance optimization of TMSs as high-performance anode materials in LIBs.

## Experimental Section

### Materials and Synthesis

Cu_2_TSiS_4_ (T = Fe, Mn) were prepared via a high-temperature solid-state reaction. High-purity Cu (99.999% mass fraction, Alfa Aesar), Si (99.999% mass fraction, Alfa Aesar), and S (99.5% mass fraction, Alfa Aesar) powders with either Mn (99.95% mass fraction, Alfa Aesar) or Fe (99.99% mass fraction, Alfa Aesar) powders were ground in an argon-filled glovebox (H_2_O < 0.1 ppm and O_2_ < 0.1 ppm) and pressed into a 6 mm pellet. The pellet was sealed in a quartz ampule under vacuum (<10^–3^ Torr), which was annealed within a muffle furnace at 600 °C for 1 d, followed by 900 °C for 3 d with a heating and cooling rate of 100 and 150 °C/h for both samples.

Single layer graphene and N-doped single layer graphene were purchased from ACS Materials, and carbon black (Super S, 99+%) was received from Alfa Aesar. Lithium hexafluorophosphate (LiPF_6_), ethylene carbonate (≥99%), diethyl carbonate (≥99%), and fluoroethylene carbonate (≥99%) were obtained from Sigma-Aldrich.

### Powder X-ray Diffraction (XRD)

The room-temperature powder X-ray diffraction (XRD) patterns were recorded using a Rigaku MiniFlex-600 benchtop powder diffractometer with Cu Ka (λ = 1.5418 Å) radiation. The general XRD patterns were collected for 1 h by increasing the scattering angle 2Θ from 10 to 90°. The data used for Rietveld refinement was collected overnight with the scattering angle 2Θ from 5 to 120°. Rietveld refinements of XRD data were carried out using the suite of FullProf programs.^39^

### Scanning Electron Microscopy (SEM)

Energy-dispersive X-ray microanalysis (EDS) and scanning electron microscope (SEM) images were collected by a JEOL JSM-IT500HRLV with an Octane Elect Plus EDS spectroscopy system. The sample was prepared on carbon tape, and an accelerating voltage of 15 kV was used. The cycled electrodes were at a fully charged stage when the SEM/EDS images were taken. SEM images of cross sections were obtained by tilling the SEM stage to an angle of 88.5°.

### Raman Spectroscopy

The Raman spectra were recorded with a Horiba XploRa PLUS Raman microscope with a 532 nm laser source. The Raman shift range was set between 50 and 3500 cm^–1^. Acquiring and accumulation times were set at 10 and 30 s, respectively.

### X-ray Photoelectron Spectroscopy (XPS)

XPS measurement was conducted with a PHI 5000 VersaProbe II system (Physical Electronics) spectrometer, which is equipped with a hemispherical analyzer. Electrodes were prepared in different states for characterization experiments. Electrochemically cycled electrodes were prepared by performing cycling at 200 mA g^–1^. Once the targeted state was reached, the coin cells were immediately disassembled, and the electrode surface was cleaned by rinsing with 3 mL of dimethyl carbonate (DMC). This rinsing process was repeated three times, with each immersion lasting 3 h, and the final rise extended overnight. Following the rinsing step, the electrodes were dried under a vacuum.

### UV–vis Spectroscopy

The UV–vis spectroscopy measurement was conducted on a Shimadzu UV-2600 Plus Spectrometer. The electrodes were immersed in 1 mL of electrolyte solution overnight. This solution was transferred into a cuvette, which was completely sealed to prevent oxidation of the electrolyte solution during analysis.

### Electrochemical Measurement

Cu_2_FeSiS_4_ (or Cu_2_MnSiS_4_) and N-doped single layer graphene (NGr) were ground with a mass ratio of 3:1 for 1 h to fabricate a homogeneous mixture. Ten wt % of carbon black (CB; Super P, >99%) was ground with the mixture for another hour. Subsequently, 10 wt % poly(vinylidene fluoride) (PVDF) in an *N*-methyl-2-pyrrolidone (NMP) solution (10 mg mL^–1^) was added to the mixture to form a slurry. The slurry was cast onto a copper film using a doctor blade. The film was then placed in an oven at 60 °C for 6 h and then in a vacuum oven at 80 °C overnight. The film was punched into circular electrodes. The average mass loading of the active material in the electrode was 1.1 mg cm^–2^. Additionally, Cu_2_FeSiS_4_–CB-PVDF and NGr-PVDF electrodes were prepared in ratios of 6:3:1 and 9:1, respectively. The Cu_2_MnSiS_4_ electrode was fabricated under the same conditions as those for the Cu_2_FeSiS_4_ electrode. The LiFePO_4_ electrode was employed for the full cell test. The LiFePO_4_ electrode was prepared by using LiFePO_4_, ketjen black (KB), and poly(tetrafluoroethylene) (PTFE) binder with a mass ratio of 8:1:1. The average mass loading of the LiFePO_4_ electrode was 5.0 mg cm^–2^. The electrolyte was prepared in the glovebox with water and oxygen contents below 0.1 ppm. 1 M lithium hexafluorophosphate (LiPF_6_) in ethylene carbonate (EC)/diethyl carbonate (DEC) with a volumetric ratio of 1:1 and 10 vol % fluoroethylene carbonate (FEC) was used as the electrolyte. Both half-cells and full cells were assembled and tested using coin-cell-type batteries. Lithium metal was used as the counter electrode, and the polypropylene (PP) membrane was employed as the separator. The entire assembling process was performed in a glovebox.

The electrochemical performance test was proceeded through Arbin (LBT20084, Arbin Instruments) and Landt (CT3002 AU-5 V10 mA, Landt Instrument) systems. Cyclic voltammograms (CV) were recorded through a Gamry Reference 1010E Potentiostat/galvanostat/ZRA with a scan rate of 0.1–1.0 mV s^–1^. Electrochemical impedance spectrometry (EIS) was also tested using a Gamry Reference 1010E Potentiostat/Galvanostat/ZRA. We hypothesized that lithiation occurred solely through a conversion reaction between Li ions/electrons and Cu_2_FeSiS_4_. Li_2_S is a major product in the reaction, while the other elements, such as Fe, Cu, and Si, will function as catalysts for the electrochemical reaction. Therefore, we calculated the theoretical specific capacity using the equation: Capacity = (*n* × *F*)/*M*_W_, where *n* is the number of lithium ions/electrons in the reaction, *F* represents the Faraday constant, and *M*_W_ is the molecular weight of the material. Based on this, the theoretical specific capacity of Cu_2_FeSiS_4_ is 631.88 mAh g^–1^.

## Results and Discussion

**Figure 2:**
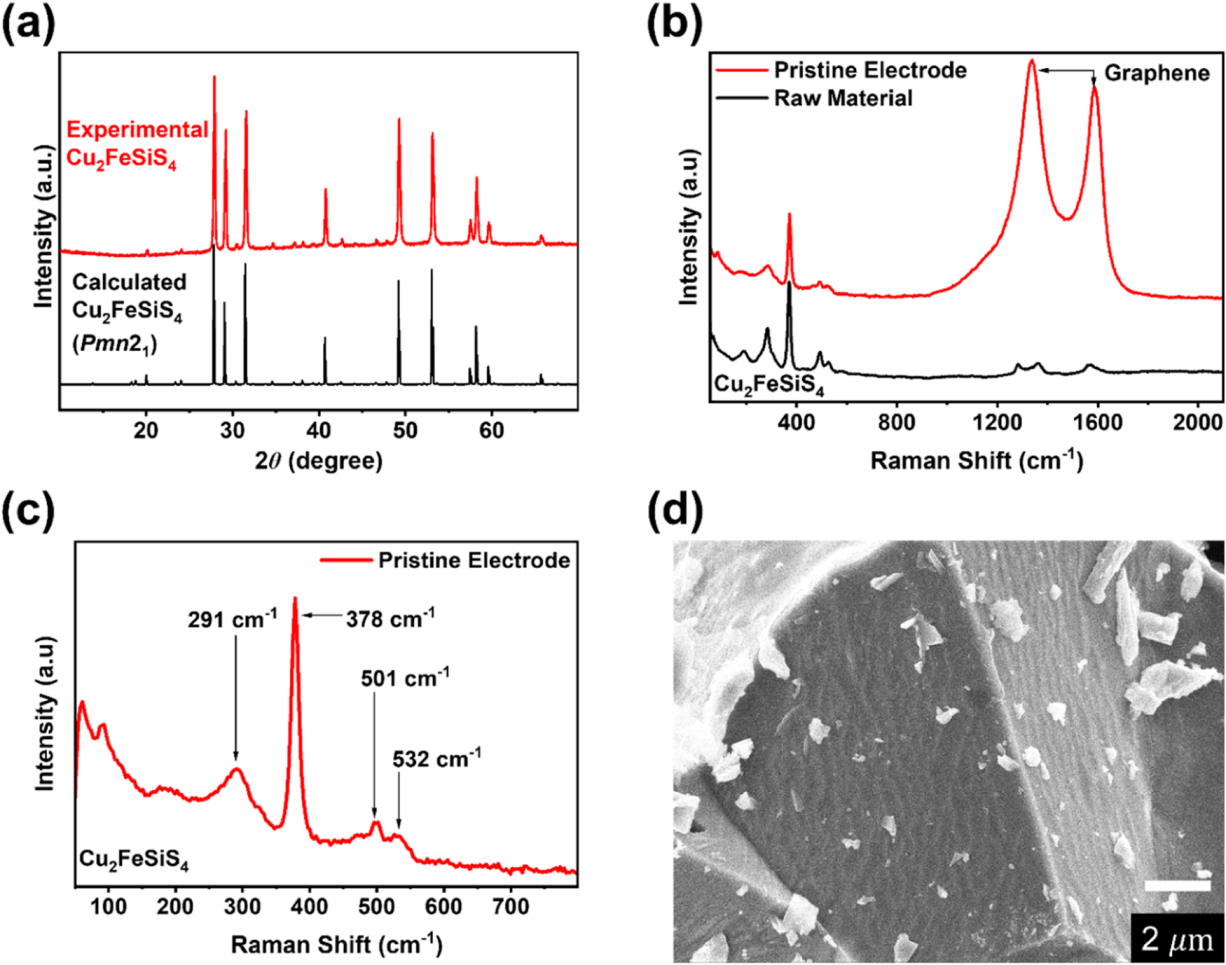
(a) Comparison of calculated and experimental XRD patterns of Cu_2_FeSiS_4_, (b) Raman spectra for the raw material and the pristine electrode in a wide range, (c) Raman spectrum for Cu_2_FeSiS_4_ in a short-range, and (d) SEM image of Cu_2_FeSiS_4_.

Quaternary TMSs, Cu_2_TSiS_4_ (T = Fe, Mn), were prepared through a high-temperature solid-state method based on our previous report.^40^ Powder XRD was utilized to study the crystal structure of these TMSs, confirming that the experimental patterns match the theoretical pattern with the space group *Pmn*2_1_ (Figures 2a and S1a). We also performed Rietveld refinements to refine the crystal structure of Cu_2_TSiS_4_ (T = Fe, Mn) using the powder XRD data that were collected overnight. The data can fit well with the orthorhombic crystal structure with the space group *Pmn*2_1_, and the Cu_2_MnSiS_4_ sample shows a small amount of Cu_2_SiS_3_ impurity (wt % = 1.3%) (Figure S2). The refined unit cell and selected parameters (Table S1) are similar to the previously reported values.^40^

The XRD pattern of the Cu_2_FeSiS_4_ electrode also corresponds to the patterns of the polycrystalline powder. In addition, Raman spectra reveal a similarity between the raw material and the pristine electrode (Figure 2b). In the pristine Cu_2_FeSiS_4_ and Cu_2_MnSiS_4_ electrodes, three peaks at 1282, 1360, and 1572 cm^–1^ are merged with the D band (disordered carbon) and G band (graphitic carbon) from NGr and CB. In the 200–600 cm^–1^ region, low-intensity peaks are observed at 291, 378, 501, and 532 cm^–1^ (Figure 2c), indicating bonds for Cu–S, Fe–S, and Si–S.^41−43^ In the Raman spectrum of Cu_2_MnSiS_4_, peaks are observed at 247.4, 285.2, 385.4, and 465.9 cm^–1^, demonstrating bonds between transition metals or silicon and sulfur (Figure S1b). The SEM images exhibit the morphologies of Cu_2_FeSiS_4_ (Figure 2d) and Cu_2_MnSiS_4_ (Figure S1c,d), both of which consist of particles with a size larger than 10 μm. The EDS elemental mapping results confirm the uniform distribution of Cu, Fe, Mn, Si, and S elements in the synthesized TMSs (Figures S3 and S4). Overall, the material characterization results validate the crystal structure and morphology of Cu_2_TSiS_4_ (T = Fe, Mn).

**Figure 3:**
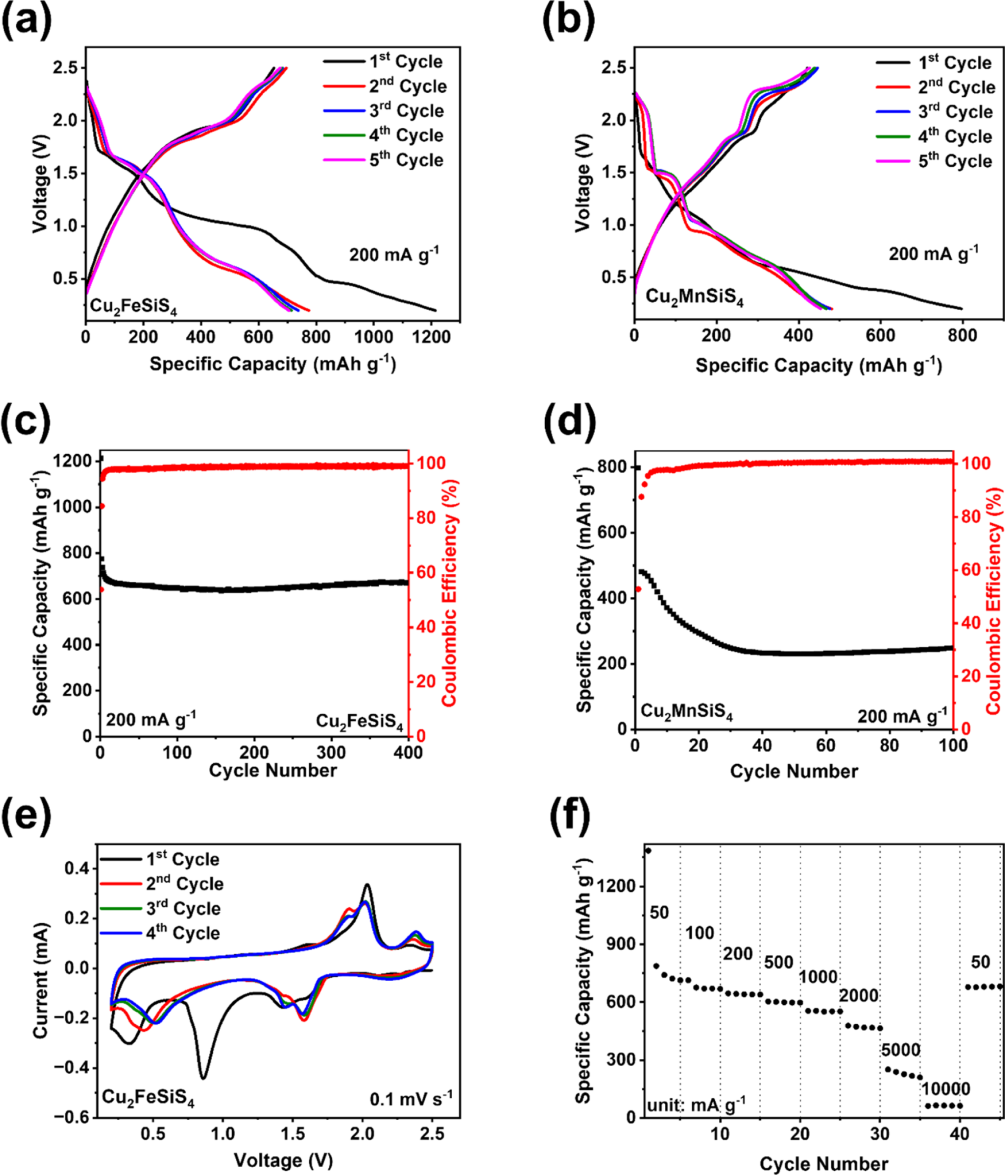
Galvanostatic charge–discharge curves for (a) Cu_2_FeSiS_4_ and (b) Cu_2_MnSiS_4_ in LIBs; cycling performance of (c) Cu_2_FeSiS_4_ and (d) Cu_2_MnSiS_4_ at 200 mA g^–1^; (e) cyclic voltammograms of Cu_2_FeSiS_4_ at 0.1 mV s^–1^; and (f) rate capability of Cu_2_FeSiS_4_ at various current densities.

The electrochemical performances of Cu_2_FeSiS_4_ and Cu_2_MnSiS_4_ were evaluated by galvanostatic charge–discharge, cyclic voltammetry (CV), electrochemical impedance spectrometry (EIS), and galvanostatic intermittent technique (GITT) tests. The charge–discharge curves of Cu_2_FeSiS_4_ and Cu_2_MnSiS_4_ at the current density of 200 mA g^–1^ are shown in Figures 3a,b, respectively. The sloping discharge plateaus of Cu_2_FeSiS_4_ are located at 2.2, 1.5, and 0.7 V, respectively, while the sloping discharge plateaus of Cu_2_MnSiS_4_ are located at 2.1, 1.5, and 0.5 V, respectively. The first discharge capacity of Cu_2_FeSiS_4_ is 1213.4 mAh g^–1^ with an initial Coulombic efficiency (CE) of 54%. In the second cycle, the discharge capacity decreases to 774.1 mAh g^–1^ with a CE of 97.2%. The initial discharge capacity of Cu_2_MnSiS_4_ is 796.6 mAh g^–1^ with a CE of 53%, and the second-cycle capacity is 475.0 mAh g^–1^. The large irreversible capacity of Cu_2_FeSiS_4_ and Cu_2_MnSiS_4_ in the first cycle is due to the growth of the solid electrolyte interphase (SEI), and irreversible conversion reactions occur during the first discharge. The long-term cycling results of Cu_2_FeSiS_4_ and Cu_2_MnSiS_4_ are shown in Figure 3c,d. The specific capacity of Cu_2_MnSiS_4_ keeps decreasing to 230 mAh g^–1^ after 50 cycles and remains stable afterward. In contrast, the reversible capacity of Cu_2_FeSiS_4_ reduces to 681.7 mAh g^–1^ at the 10th cycle and remains stable afterward.

After 400 cycles, the discharge capacity is still retained at 670.3 mAh g^–1^ (Figure 3c). Cu_2_FeSiS_4_ exhibits a better electrochemical performance than Cu_2_MnSiS_4_ in terms of specific capacity and cycle life, so further electrochemical measurements are focused on Cu_2_FeSiS_4_. The electrochemical performance of Cu_2_FeSiS_4_ was also evaluated in the cutoff voltage window of 1.0–3.0 V. As shown in Figure S5, the specific capacity of Cu_2_FeSiS_4_ in this cutoff voltage window is much lower than that observed in the cutoff voltage window of 0.2–2.5 V. Its discharge plateau is centered at ∼1.6 V, which is low for the cathode. It also suffers from a fast capacity loss in the initial 20 cycles. Therefore, Cu_2_FeSiS_4_ composite is insufficient to function as a cathode material in LIBs. The electrochemical performances of the Cu_2_FeSiS_4_ electrode without NGr at 200 mA g^–1^ are exhibited in Figure S6a,b. The initial specific capacity is 101.3 mAh g^–1^ with a Coulombic efficiency of 99.6%. Furthermore, the specific capacity is quickly reduced in the initial 35 cycles. The specific capacity is decreased to 20.2 mAh g^–1^ at the 200th cycle. The performances of the NGr-PVDF electrode are shown in Figure S6c,d. The NGr electrode initially delivers a specific capacity of 482 mAh g^–1^ with a Coulombic efficiency of 17.7%, which decreases to 54.2 mAh g^–1^ with a Coulombic efficiency of 98.85% after 200 cycles. Therefore, incorporating NGr into the Cu_2_FeSiS_4_ composite significantly improves the electrochemical performance and stability of the Cu_2_FeSiS_4_ electrode.

In the CV test (Figure 3e), the Cu_2_FeSiS_4_ electrode exhibits reduction peaks at 1.6, 1.4, 0.9, and 0.3 V, as well as oxidation peaks at 1.6, 2.0, and 2.3 V in the first cycle. The sharp reduction peak at 0.9 V in the first cycle is attributed to the formation of the SEI layer, and it disappears in the following cycles. In the CV test of Cu_2_MnSiS_4_, reduction peaks are observed at 0.7, 1.0, 1.4, 2.0, and 2.2 V in the first cycle, and oxidation peaks exhibit at 1.4 and 2.1 V (Figure S7a). The reduction peak at 1.0 V in the first cycle is also attributed to the formation of the SEI layer. The rate capability test of Cu_2_FeSiS_4_ is shown in Figure 3f. The test was performed at various current densities from 50 mA g^–1^ to 10 A g^–1^ for 5 cycles at each current density. The reversible capacity at high current densities of 5 and 10 A g^–1^ was recorded as 235 and 65 mA g^–1^, respectively. When the current density was returned to 50 mA g^–1^, the reversible capacity recovered to 680 mA g^–1^ immediately, corresponding to 93.8% of the initial capacity and demonstrating robust reaction kinetics. Therefore, the electrochemical results confirm that Cu_2_FeSiS_4_ is a promising anode material for LIBs.

**Figure 4:**
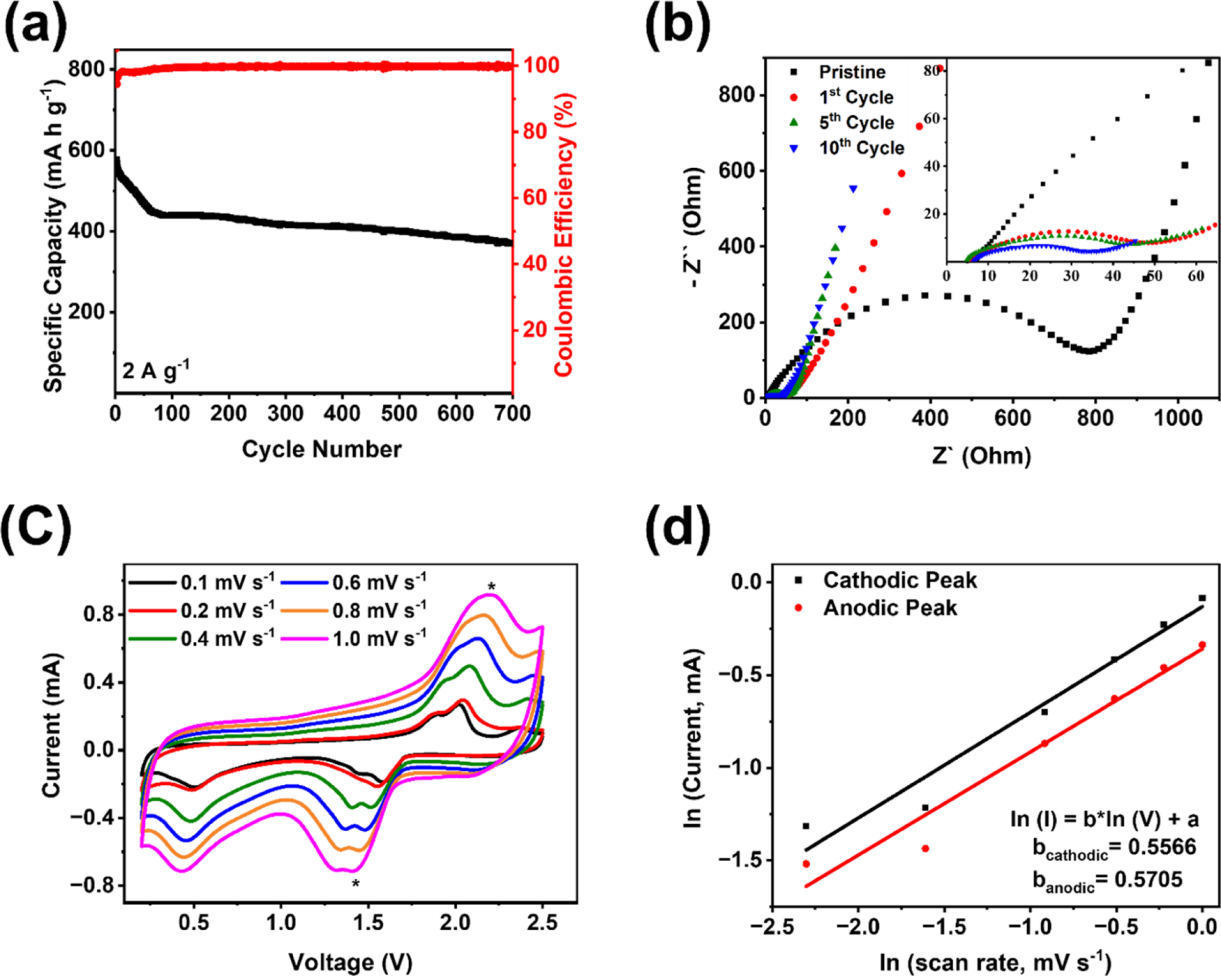
(a) Long cycling performance of Cu_2_FeSiS_4_ at 2 A g^–1^; (b) impedance analysis of Cu_2_FeSiS_4_ at different cycles; (c) cyclic voltammograms at various scan rates; (d) natural logarithm of peak current versus scan rate.

To further exploit the kinetic performance of Cu_2_FeSiS_4_, high-current-density galvanostatic charge–discharge tests EIS, GITT, and CV at various scan rates were employed. The long-term cycling test of Cu_2_FeSiS_4_ was performed at a high current density of 2 A g^–1^ to verify the fast-charging capability (Figures 4a and S7b). It delivers a reversible capacity of 370.1 mAh g^–1^ with a CE of 99.8% after 700 cycles, confirming its high cyclic stability and fast-charging capability. The charge–discharge curves at a current density of 2 A g^–1^ are provided in Figure S8. These curves exhibit a slight reduction in specific capacity, suggesting that the composite electrode exhibits a high cyclic stability with minimal capacity loss.

In Figure 4b, the depressed semicircles in the EIS data represent the interfacial resistance between Cu_2_FeSiS_4_ and the electrolyte. The resistance value of the pristine anode is 777.5 Ω. This resistance significantly decreases to 39.3 Ω after the first cycle and gradually decreases to 39 and 27.4 Ω after five and ten cycles, respectively. A small interfacial resistance indicates the formation of a stable and robust SEI layer during the initial cycles. Various scan rates from 0.1 to 1.0 mV s^–1^ were used for CV tests (Figure 4c), and these data were applied to the natural logarithm of peak current and scan rates (Figure 4d). The slopes of the cathodic and anodic peaks are 0.5566 and 0.5705, respectively. Both slopes are close to 0.5, indicating that the reaction kinetics is dominated by a diffusion-controlled process.^44,45^ The GITT results indicate overpotentials of 150 mV for charge and 110 mV for discharge, respectively (Figure S7c). According to the charge–discharge curves in GITT, the charge–discharge capacities are 708.3 and 812.3 mAh g^–1^. These results confirm that Cu_2_FeSiS_4_ exhibits fast reaction kinetics.

**Figure 5:**
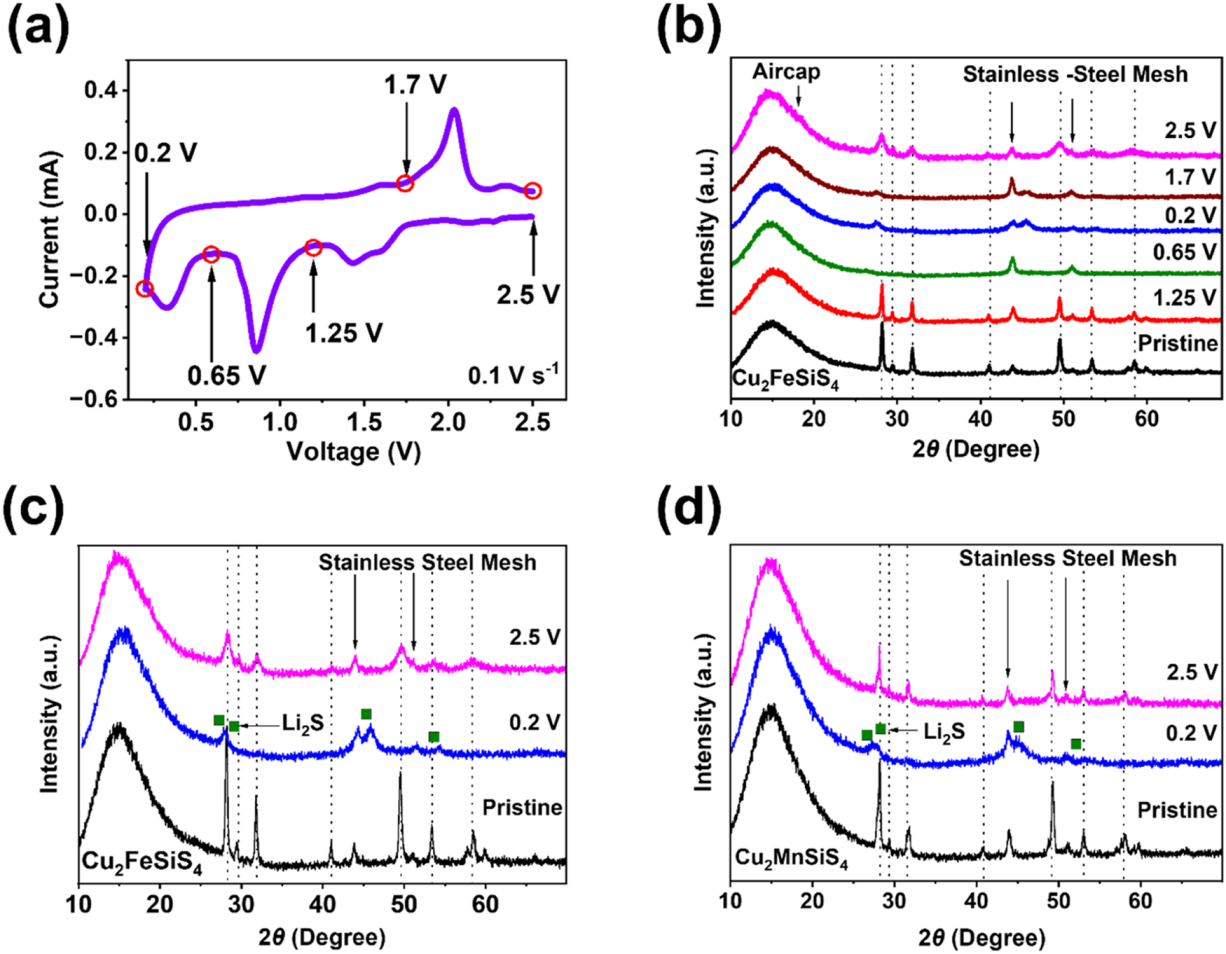
(a) Cyclic voltammogram of Cu_2_FeSiS_4_ for the 1st cycle; (b) XRD patterns of Cu_2_FeSiS_4_ at different voltages; XRD patterns of (c) Cu_2_FeSiS_4_ and (d) Cu_2_MnSiS_4_ at the pristine state, discharged to 0.2 V, and charged 2.5 V.

As a TMS, Cu_2_FeSiS_4_ undergoes conversion reactions during the lithiation and delithiation processes. However, it not only contains transition metal elements (Cu and Fe) but also includes Si (IV group), which is widely used as an alloying type of anode material.^46^ Hence, the electrochemical redox reaction mechanism of the Cu_2_FeSiS_4_ anode should be clarified. To gain insight into the reaction mechanism, XRD was utilized to exploit the crystal structure evolution of Cu_2_FeSiS_4_ before and after cycling. The Cu_2_FeSiS_4_ anodes at different charge and discharge stages during the first cycle (Figure 5a) were analyzed by XRD, as shown in Figure 5b. The broad peak from 10 to 27° was induced by the airtight cover of the XRD sample holder. The two small peaks at 43.5 and 51.1° were derived from the stainless-steel mesh. The XRD pattern of the Cu_2_FeSiS_4_ anode discharged at 1.25 V corresponds with the pristine anode.

When the Cu_2_FeSiS_4_ anode is further discharged to 0.2 V, all peaks corresponding to the Cu_2_FeSiS_4_ phase patterns of the pristine electrode disappear. Some broad peaks at 27, 31, 45, and 54° indicate the formation of Li_2_S.^47^ After being recharged back to the initial voltage stage at 1 cycle (2.5 V), most of the XRD peaks can be recovered, suggesting that the Cu_2_FeSiS_4_ anode undergoes phase changes and returns to its pristine structure after cycling (Figure 5c). We performed XRD measurements for the pristine and cycled electrodes after 5, 10, and 20 cycles. The XRD patterns of the cycled electrodes represented similarity, with small peaks detected in the 25–35° and 45–50° regions (Figure S9). The XRD patterns of the Cu_2_MnSiS_4_ anode are similar to those of the Cu_2_FeSiS_4_ anode (Figure 5d). Therefore, the XRD results indicate that the Cu_2_FeSiS_4_ anode undergoes a reversible redox reaction with Li_2_S as a major component in the products.

To understand the interfacial structure of the Cu_2_FeSiS_4_ anode, XPS measurements were performed. The XPS 1s spectra of Li, F, C, and O are analyzed in Figure S10. In XPS spectra, the chemical bonding formed on the surface of the electrode during charge and discharge can be observed, as XPS only measures the surface with a thickness of up to 10 nm. Li–F is the primary component of the SEI layer. Additional components such as RO-Li, Li_*x*_PF_*y*_, and RC-Li were also detected in the cycled electrodes. The surface XPS spectra were collected at *t* = 0 s, and the depth XPS spectra were obtained after 180 s of sputtering (Figure S11). However, determining the precise ptering thickness from XPS is challenging for the Cu_2_FeSiS_4_ electrode. This complexity arises because the sputtering rate calibrated for a Si reference cannot be directly applied to the more intricate interface of the Cu_2_FeSiS_4_ electrode. As shown in Figure S11, the content of LiF decreases from 52.6 to 25.9% in the cycled electrode after 3 min sputtering, while the content of Li_2_S increases from 24.6 to 28%. LiF is a key component in the solid electrolyte interphase. LiF content decreases because the interphase is partially removed by sputtering. On the contrary, the content of Li_2_S increases after sputtering, indicating that Li_2_S is formed in the bulk material after cycling. In addition, the formation of Li_2_S was observed after the electrode was discharged to 0.2 V, and it was retained in the following cycled electrodes, confirming a bulk conversion process of Li_2_S in the electrode.

**Figure 6:**
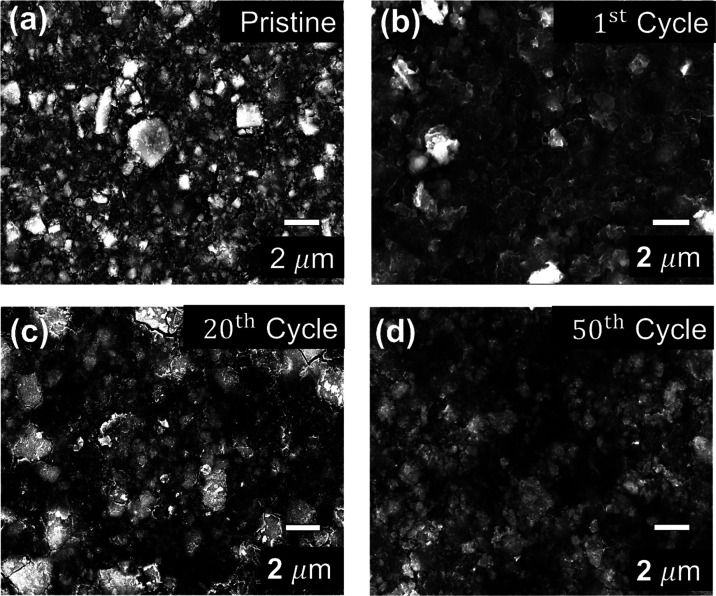
SEM images of the (a) pristine and cycled Cu_2_FeSiS_4_ anodes at (b) 1st, (c) 20th, and (d) 50th cycles.

In addition to the phase transition during cycling, the SEI layer was also spontaneously formed during the first cycle. To investigate the morphology of cycled anodes and confirm the existence and morphology of the SEI layer, SEM, EDS, and XPS experiments were performed. The SEM images of the pristine and cycled Cu_2_FeSiS_4_ anodes are presented in Figure 6. There are no obvious cracks or damages from the pristine to 50th cycled anodes, demonstrating good structure stability. EDS mapping of the first cycled electrode detected new elements, such as F, and O, which are derived from the electrolyte (Figures S12 and S13). In the XPS spectra, various SEI components are detected, such as Li_*x*_PF_*y*_, LiF, RC-Li, and RO-Li. To assess the expansion in electrode thickness through forming the SEI layer, the SEM images were captured in the *xy*-plane (Figure S14). The average thickness of the pristine electrode is 19.8 ± 4.1 μm, though some variability was noted due to the use of a doctor blade and circular punch. After the full discharge, the electrode thickness was measured at around 19.89 ± 2.6 μm. The 1- and 5-cycle electrodes exhibit the thickness of 19.9 ± 2.8 and 19.78 ± 1.4 μm, respectively. These results suggest that no significant volume expansion of the electrode from SEI formation could be detected within these early cycles.

The surface morphology of the Cu_2_MnSiS_4_ anode is exhibited in Figure S15. In the pristine anode, its morphology is similar to that of the Cu_2_FeSiS_4_ anode, but cracks and holes appeared after 50 cycles, indicating that the Cu_2_MnSiS_4_ composite is less stable than Cu_2_FeSiS_4_. Additionally, UV–vis spectroscopy was utilized to assess the stability of each electrode (Figure S16). The fully discharged and fifth cycled samples exhibited higher absorption than other samples. However, Cu_2_MnSiS_4_ represented a slightly elevated absorbance, suggesting that some components were dissolved into the electrolyte. This dissolution results in faster capacity fading and more severe surface damage in Cu_2_MnSiS_4_ electrodes compared to Cu_2_FeSiS_4_ electrodes.^48,49^

**Figure 7:**
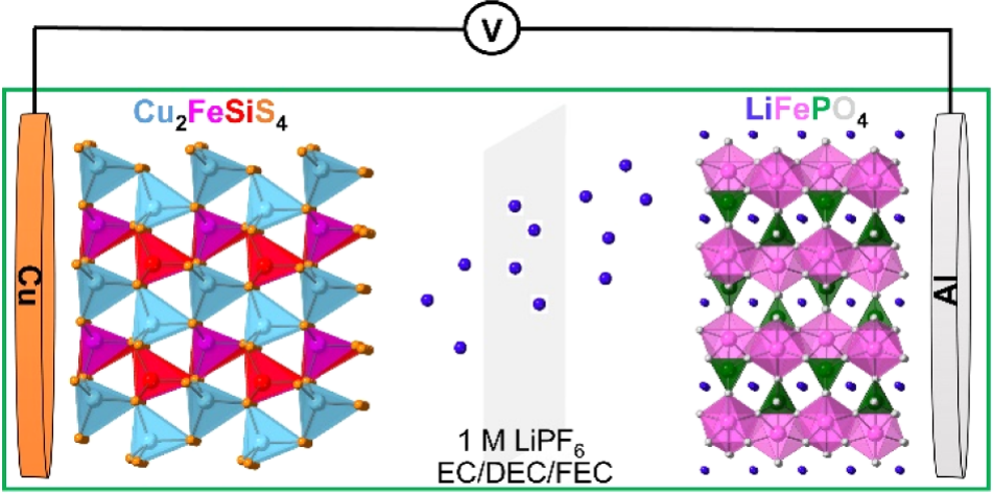
Illustration of a full cell with Cu_2_FeSiS_4_ as the anode and LiFePO_4_ as the cathode.

**Figure 8:**
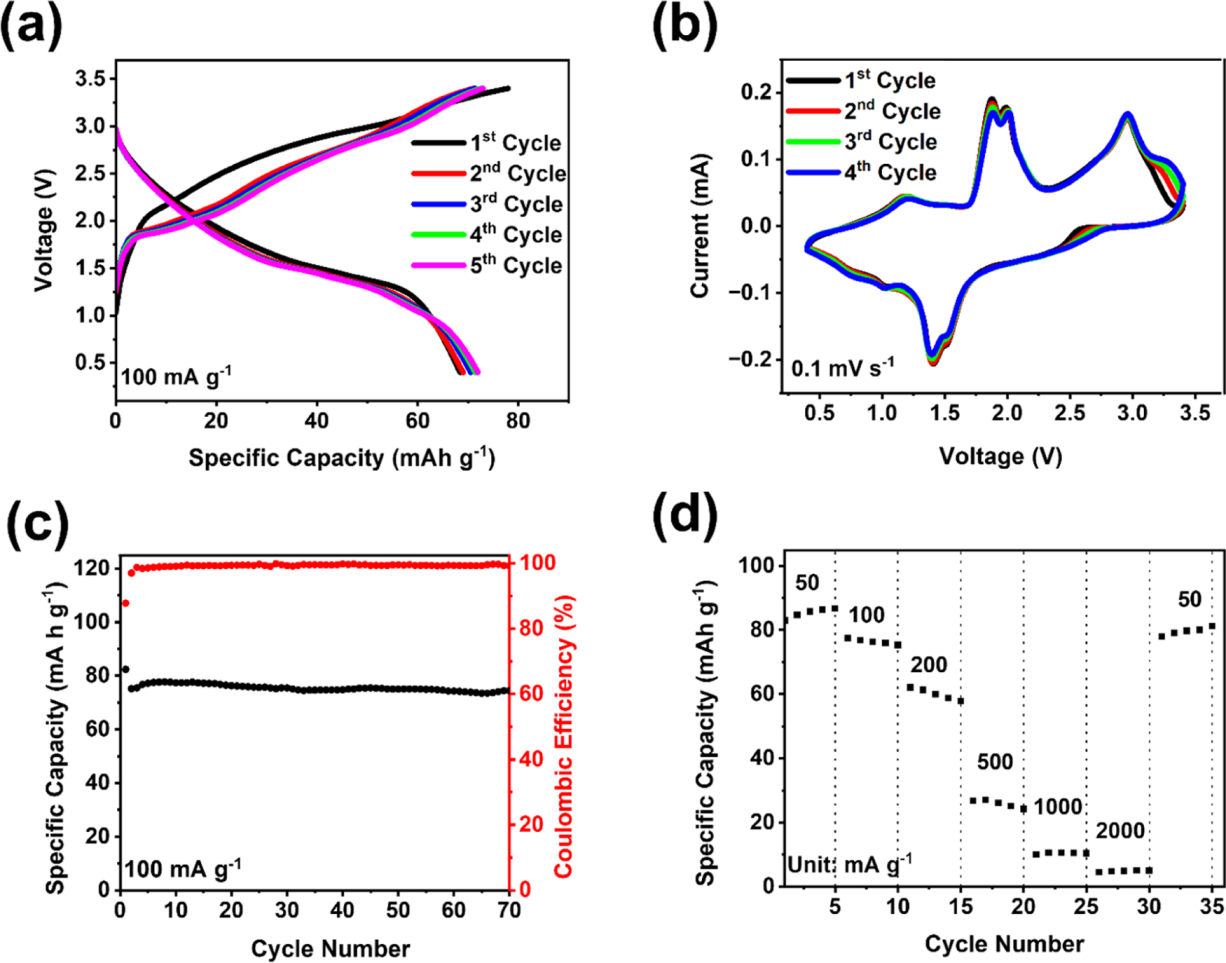
Electrochemical performance of LiFePO_4_||Cu_2_FeSiS_4_ full cells: (a) galvanostatic charge–discharge curves; (b) cyclic voltammograms at 0.1 mV s^–1^; (c) cycling performance at 100 mA g^–1^; and (d) rate capability of LiFePO_4_||Cu_2_FeSiS_4_ full cells at various current densities.

Due to the high performance of Cu_2_FeSiS_4_, it was coupled with a commercially available cathode material (LiFePO_4_) to make LiFePO_4_||Cu_2_FeSiS_4_ full cells (Figure 7). Before the full cell test, the LiFePO_4_ half-cell was assessed in 1 M LiPF_6_ in EC/DEC (1:1) with 10% FEC electrolyte, delivering exceptional electrochemical performance (Figure S17). The galvanostatic charge–discharge curves of the LiFePO_4_||Cu_2_FeSiS_4_ full cell are shown in Figure 8a. The full cell delivered a pair of sloping plateaus centered at ∼2 V with a reversible capacity of ∼70 mAh g^–1^ at 100 mA g^–1^ based on the total weight of the cathode and the anode. Cyclic voltammetry was performed at a rate of 0.1 mV s^–1^ (Figure 8b). Multiple pairs of cathodic and anodic peaks were observed in the CV, corresponding to the sloping plateaus in the galvanostatic charge–discharge curves (Figure 8a). The long-term cycling stability result in Figure 8c shows that the initial specific capacity of the full cell is 82.4 mAh g^–1^ with a CE of 87.8%. After 70 cycles, the specific capacity remains at 74.4 mAh g^–1^ with a CE of 99.4%. The rate performance of the full cell was measured under various current densities of 50, 100, 200, 500, 1000, 2000, and 50 mA g^–1^. As shown in Figure 8d, the specific capacity is 86.7 mAh g^–1^ at 50 mA g^–1^. When the current densities were increased to 500 mA g^–1^, the specific capacity was decreased to 28 mAh g^–1^. After the current density decreased back to 50 mA g^–1^, the specific capacity was immediately increased to 81.7 mAh g^–1^, demonstrating robust reaction kinetics. Therefore, the full cell test results manifest the great promise of the Cu_2_FeSiS_4_ anode in practical applications for LIBs.

## Conclusions

In summary, this work demonstrates a promising transition metal silicon sulfide-based anode material (Cu_2_FeSiS_4_) for LIBs. The material adopts an orthorhombic crystal structure with chains of corner-sharing CuS_4_, FeS_4_, and SiS_4_ tetrahedra. The unique structure of Cu_2_FeSiS_4_ enables high electrochemical performance in terms of high specific capacity, long cycle life, and fast-charging capability. Its reaction kinetics was investigated through the rate capability tests, high-current-density galvanostatic charge and discharge tests, CV under various scan rates, EIS, and GITT, indicating high-rate performance, low interfacial resistance, and a stable SEI layer. The stability and reaction mechanism were explored using SEM and XRD, demonstrating stable morphology upon cycling and the reversible redox reaction. Moreover, the Cu_2_FeSiS_4_ anode was coupled with the LiFePO_4_ cathode to construct LiFePO_4_||Cu_2_FeSiS_4_ full cells, which also delivered superior electrochemical performance. These results demonstrate great promise for quaternary transition metal silicon sulfides as anodes in low-cost and sustainable LIBs.

Polycrystalline quaternary transition metal silicon sulfides exhibited high specific capacity and long cycle life, representing promising anode materials for LIBs. However, the redox potentials of Cu_2_FeSiS_4_ and Cu_2_MnSiS_4_ are centered at ∼1 V, and their potential hysteresis is large, compromising the energy density of Li-ion full cells. To address these challenges, it is critical to increase the reaction kinetics and tune the material chemistries by doping silicon sulfides with various metal elements. Nanotechnology can be employed to reduce the particle size from microscale to nanoscale, shortening the ion diffusion pathways and increasing the surface area for more reactive sites. Carbon coating on the surface of these anode materials can increase the conductivity, facilitating ion/electron transport. These will enhance the reaction kinetics and lower the potential hysteresis. To decrease the redox potentials, various metal elements, such as Al, Ti, Sn, Ge, and so forth, can be doped into silicon sulfides to alter the HOMO and LUMO energy levels. In addition, electrolytes also play a significant role in battery performance. Various electrolytes, such as fluorinated electrolytes, high-concentration electrolytes, localized high-concentration electrolytes, high-entropy electrolytes, etc., can be used to form stable and robust interphases for high-performance silicon sulfide anodes in LIBs. The synergy of these strategies will achieve high-energy LIBs for practical applications.

## Supporting Information Available

XRD; Raman Spectra; SEM of Cu_2_MnSiS_4_; Rietveld refinements of Cu_2_TSiS_4_ (T = Fe, Mn); EDS elemental maps of Cu_2_TSiS_4_ (T= Fe, Mn); electrochemical performance of Cu_2_TSiS_4_ (T = Fe, Mn); EDS elemental maps and elemental analysis of cycled of Cu_2_FeSiS_4_; SEM images of Cu_2_MnSiS_4_ anode; XPS spectra of Cu_2_FeSiS_4_ anode; cross-sectional SEM images; solubility test for composite electrodes in the electrolyte; and cyclic performance of LiFePO_4_ (PDF)
