# Supplementary material for: Quaternary Cu2TSiS4 (T = Fe, Mn) Anodes for Li-Ion Batteries
Source: ACS Appl Energy Mater. 2025 Jan 18;8(3):1908–17. doi: 10.1021/acsaem.4c03366 (PMC11815620; doi:10.1021/acsaem.4c03366)
Supplement: Supplementary file 1 — ae4c03366_si_001.pdf [file ae4c03366_si_001.pdf]

Quaternary Cu_2TSiS_4 (T = Fe, Mn) Anodes for Li-ion Batteries

Eric Youngsam Kim,[1] Zachary T. Messegee,[1] Zhenzhen Yang[2], Xiaoyan Tan,[1,3*] Chao Luo[1,3,4*]

[1]*Department of Chemistry and Biochemistry, George Mason University, Fairfax, Virginia 22030, USA*

[2]*Chemical Sciences and Engineering Division, Argonne National Laboratory, Lemont, IL 60439, USA*
[3]*Quantum Science & Engineering Center, George Mason University, Fairfax, Virginia 22030, USA*
[4]*Department of Chemical, Environmental and Materials Engineering, University of Miami, Coral Gables, FL 33146, USA*
**Corresponding Author E-mails: cxl1763@miami.edu; xtan6@gmu.edu*

Page

Figure S1. XRD, Raman spectra, and SEM of Cu_2MnSiS_4 .... S2
Figure S2. Rietveld refinements of Cu_2FeSiS_4 (a) and Cu_2MnSiS_4 (b) in the space group *Pmn*2_1 using room-temperature powder XRD data S2
Table S1. Selected Selected Structure Parameters and Refinement Details for Cu_2TSiS_4 (T = Fe, Mn) using Powder XRD Data S3
Figure S3. EDS elemental maps of polycrystalline Cu_2FeSiS_4 .... S3
Figure S4. EDS elemental maps of polycrystalline Cu_2MnSiS_4 .... S4
Figure S5. Electrochemical performance of Cu_2FeSiS_4/Ngr composite electrode in the cutoff voltage window of 1.0-3.0 V. (a) Galvanostatic charge-discharge curves at 200 mA g^{-1}; (b) cycling performance at 200 mA g^{-1}; (c) cyclic voltammetry at 0.4 mV s^{-1} S4
Figure S6. Electrochemical performances of Cu_2FeSiS_4 electrode without NGr in LIBs S4
Figure S7. a) Cyclic Voltammograms of Cu_2MnSiS_4 at 0.1 mV s^{-1}; (b) galvanostatic charge-discharge curves of Cu2FeSiS4 at 2 A g^{-1} (c) Galvanostatic intermittent titration technique (GITT) test for the Cu_2FeSiS_4 anode at 200 mA g^{-1} S5
Figure S8. Galvano-static charge-discharge spectrum for Cu_2FeSiS_4 composite anode at 2 A g^{-1}. S5
Figure S9. XRD patterns of Cu_2FeSiS_4 anodes at different numbers of cycles S5
Figure S10. Li 1s, F 1s, C1s, and O1s XPS spectra of Cu_2FeSiS_4 anode at various charge and discharge status...... S6
Figure S11. Li 1s, F 1s, C1s, and O1s XPS surface and depth spectra of Cu_2FeSiS_4 anode at 1st cycle and 5th cycles. S7
Figure S12. EDS elemental maps of the 1 cycled Cu_2FeSiS_4 anode. S8
Figure S13. EDS elemental analysis of the cycled Cu_2FeSiS_4 anode S8
Figure S14. Cross-sectional SEM images. (a) pristine; (b) first fully discharged; (c) 1st cycled; (d) 5th cycled electrode S9
Figure S15. SEM images of the (a) pristine and (b,c) cycled Cu_2MnSiS_4 anodes S9
Figure S16. Solubility test for composite electrodes in the electrolyte, 1M LiPF6 in EC:DEC (1:1/V) with FEC (10%). UV-vis spectra of electrolyte. (a) Cu_2FeSiS_4; (b) Cu_2MnSiS_4. .... S10
Figure S17. (a) Galvanostatic charge-discharge curves of $LiFePO_4$ in 1M LiPF6 in EC:DEC (1:1 /V) with 10 % FEC; (b) Cycle life and Coulombic efficiency of $LiFePO_4$ half-cell upon long-term cycling. S10

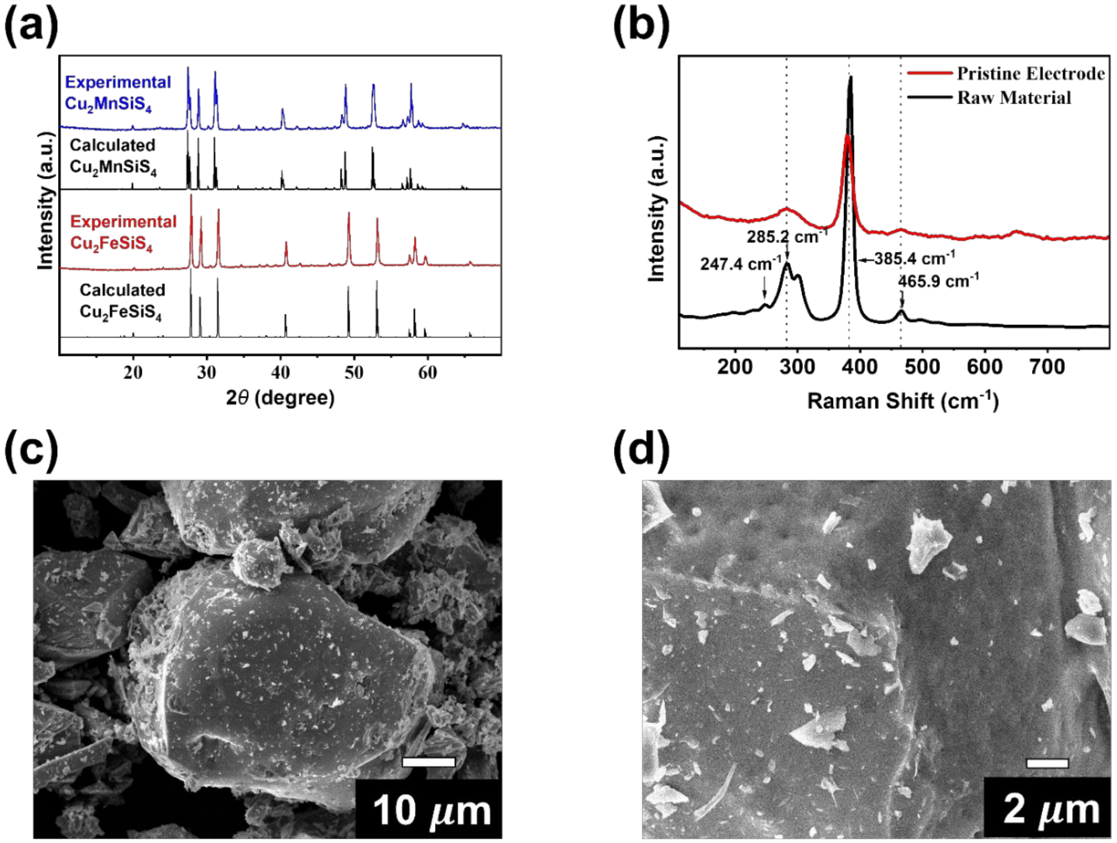

Figure S1. (a) Experimental and theoretical XRD patterns of Cu_2FeSiS_4 and Cu_2MnSiS_4; (b) Raman spectra of polycrystalline Cu_2MnSiS_4 and the anode; (c-d) SEM images of polycrystalline Cu_2MnSiS_4.

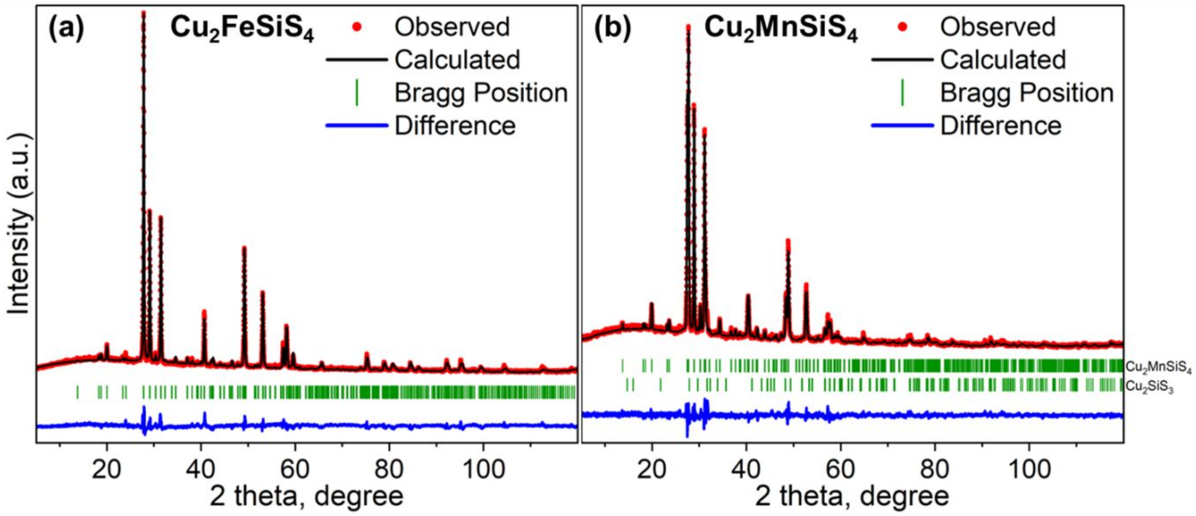

Figure S2. Rietveld refinements of Cu_2FeSiS_4 (a) and Cu_2MnSiS_4 (b) in the space group *Pmn*2_1 using room-temperature powder XRD data. The observed data (red), calculated pattern (black), expected Bragg peak positions (green), and the difference between observed and calculated (blue) are shown.

Table S1. Selected Structure Parameters and Refinement Details for Cu_2TSiS_4 (T = Fe, Mn) using Powder XRD Data.

sample		Cu_2FeSiS_4	Cu_2MnSiS_4
temperature		300 K	300 K
mol. wt., g/mol		339.28	338.38
density (calculated), g/cm^3		3.870	3.761
x-ray wavelength		1.54056 Å	1.54056 Å
space group, #		*Pmc*2_1, # 36	*Pmc*2_1, # 36
Z		2	2
lattice parameters		a = 7.4014(2) Å,<br>b = 6.4138(1) Å,<br>c = 6.1329(1) Å,<br>V = 291.14(1) Å^3	a = 7.5213(1) Å,<br>b = 6.4328(1) Å,<br>c = 6.1760(1) Å,<br>V = 298.81(1) Å^3
Rietveld criteria of fit		R_p = 5.35 %, R_{wp} = 6.91%,<br>R_{exp} = 4.37%, χ^2 = 2.49	R_p = 4.92%, R_{wp} = 6.42%,<br>R_{exp} = 5.61%, χ^2 = 1.31
site	Wyckoff symbol	*x, y, z*	
Cu1	4b	0.2553(6), 0.3289(3), 0.025(1)	0.7489(7), 0.6811(6), 0.186(5)
Fe1/Mn1	2a	0, 0.1365(6), 0.5	0, 0.8333(6), 0.689(5)
Si1	2a	0, 0.827(1), 0	0, 0.232(2), 0.162(5)
S1	2a	0, 0.836(1), 0.353(1)	0, 0.8467(7), 0.107(5)
S2	2a	0, 0.169(1), 0.926(1)	0, 0.1899(6), 0.386(5)
S3	4b	0.235(1), 0.3136(7), 0.393(1)	0.7325(7), 0.6926(7), 0.574(5)

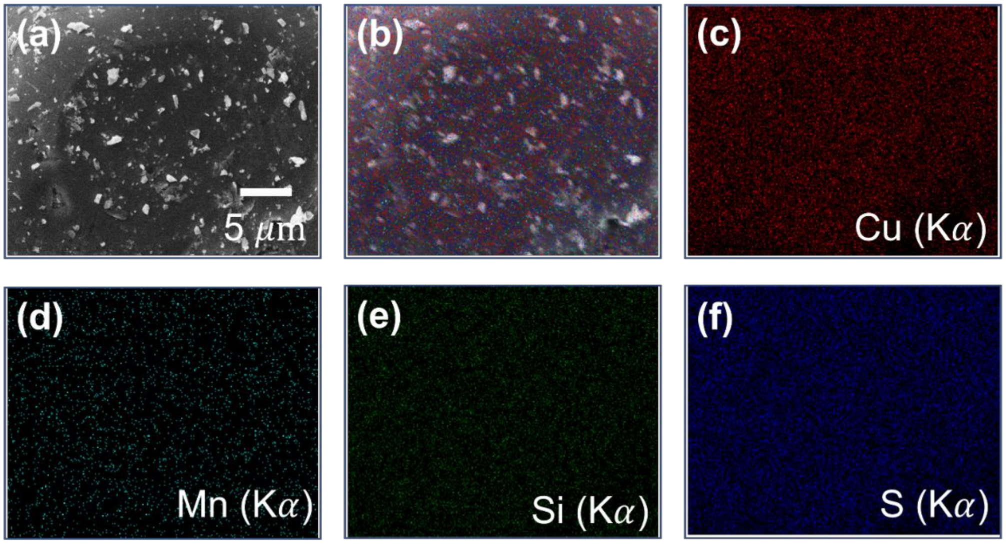

Figure S3. (a-f) EDS elemental maps of polycrystalline Cu_2FeSiS_4.

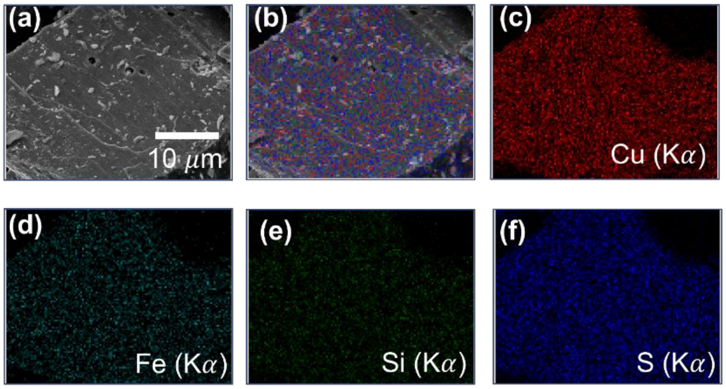

Figure S4. (a-f) EDS elemental maps of polycrystalline Cu_2MnSiS_4.

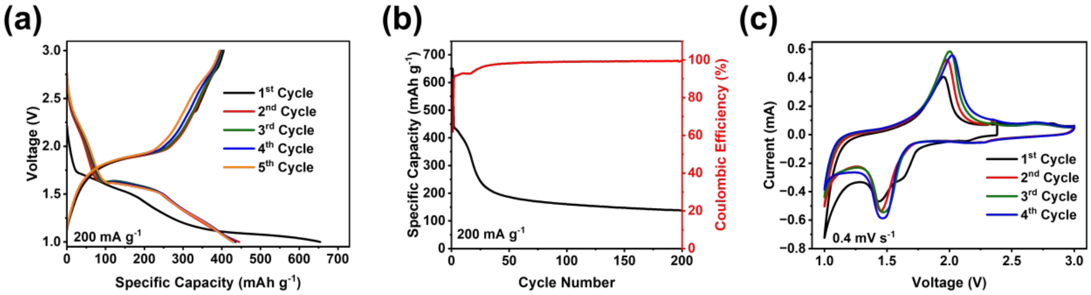

Figure S5. Electrochemical performance of Cu_2FeSiS_4/Ngr composite electrode in the cutoff voltage window of 1.0-3.0 V. (a) Galvanostatic charge-discharge curves at 200 mA g^{-1}; (b) cycling performance at 200 mA g^{-1}; (c) cyclic voltammetry at 0.4 mV s^{-1}.

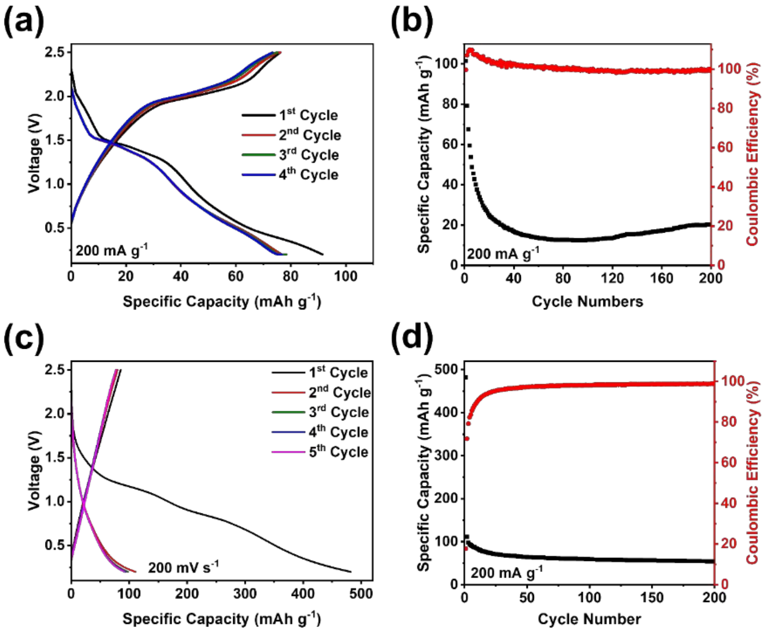

Figure S6. Electrochemical performances of Cu_2FeSiS_4 electrode without NGr in LIBs. (a) Galvanostatic charge-discharge curves at 200 mA g^{-1}; (b) cycling performances at 200 mA g^{-1}; NGr electrode with CB and PVDF (6:3:1 ratio). (c) Galvanostatic charge-discharge curves at 200 mA g^{-1}; (d) cycling performance at 200 mA g^{-1}.

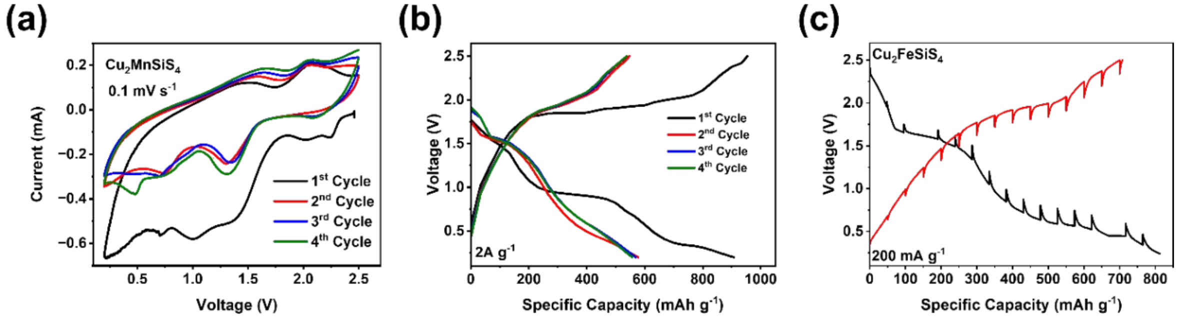

Figure S7. (a) Cyclic Voltammograms of Cu_2MnSiS_4 at 0.1 mV s^{-1}; (b) galvanostatic charge-discharge curves of Cu_2FeSiS_4 at 2 A g^{-1} (c) Galvanostatic intermittent titration technique (GITT) test for the Cu_2FeSiS_4 anode at 200 mA. g^{-1}.

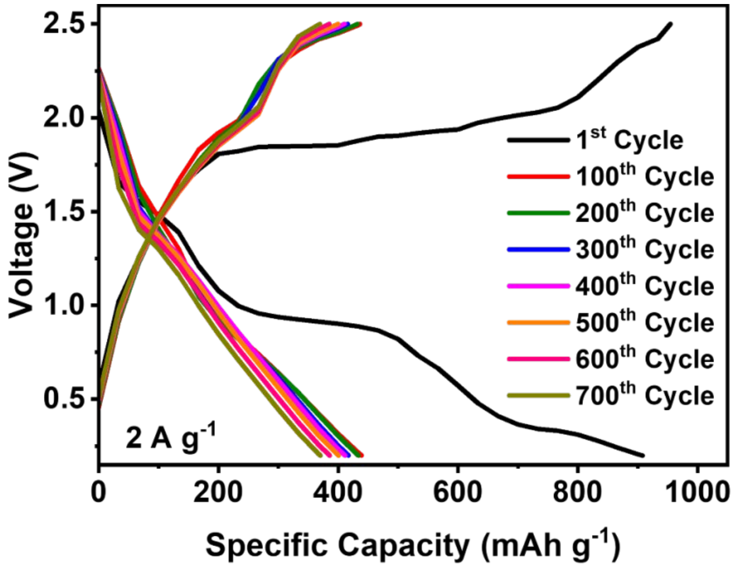

Figure S8. Galvano-static charge-discharge spectrum for Cu_2FeSiS_4 composite anode at 2 A g^{-1}.

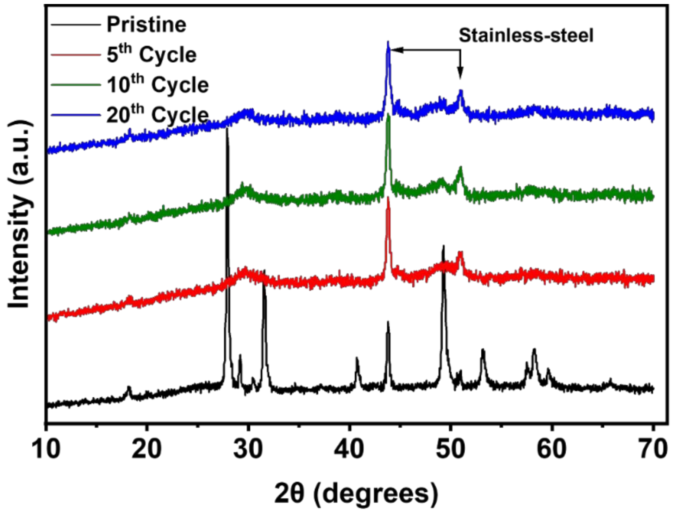

Figure S9. XRD patterns of Cu_2FeSiS_4 anodes at different numbers of cycles.

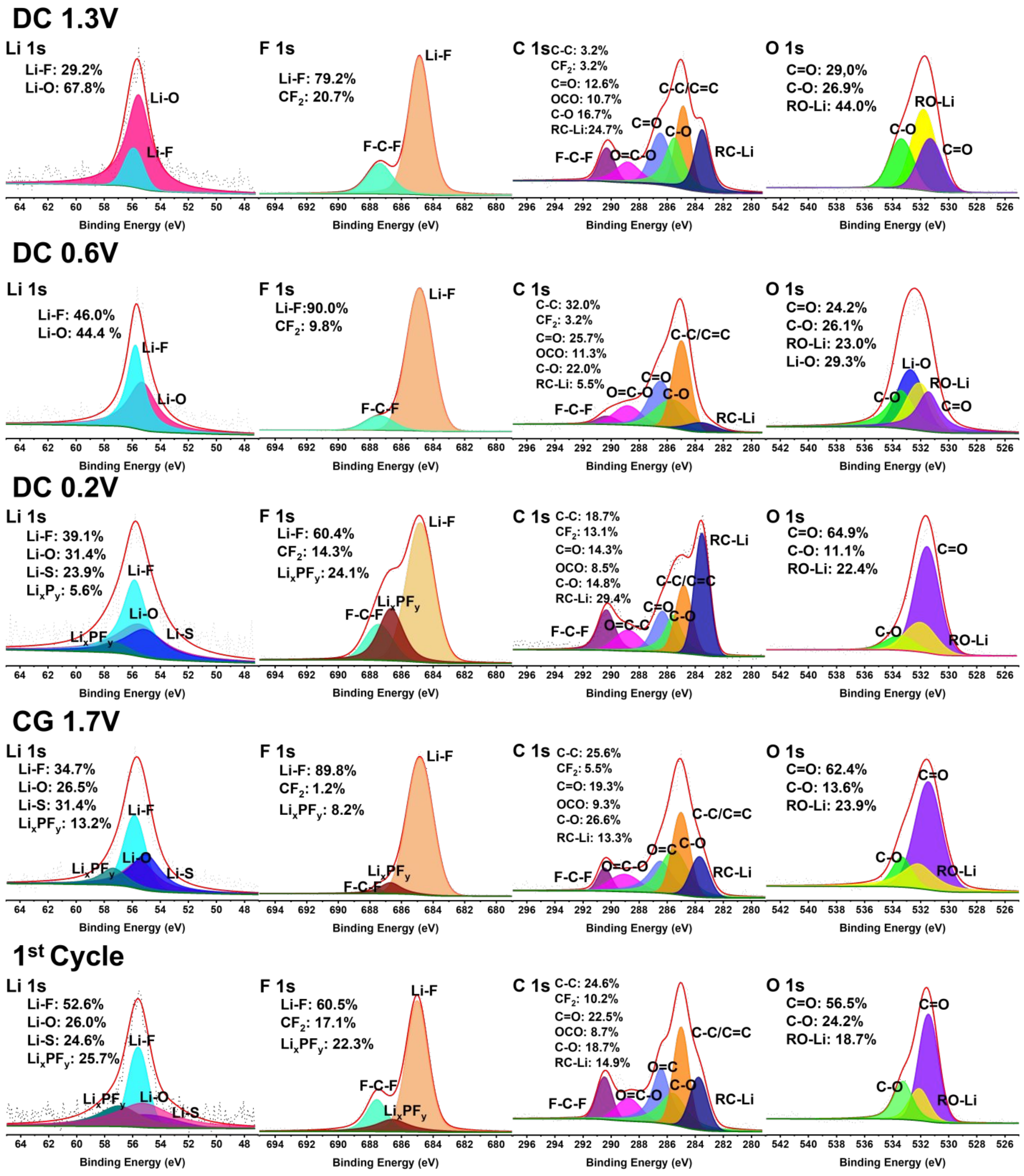

Figure S10. Li 1s, F 1s, C1s, and O1s XPS spectra for the Cu_2FeSiS_4 anode at various charge and discharge status.

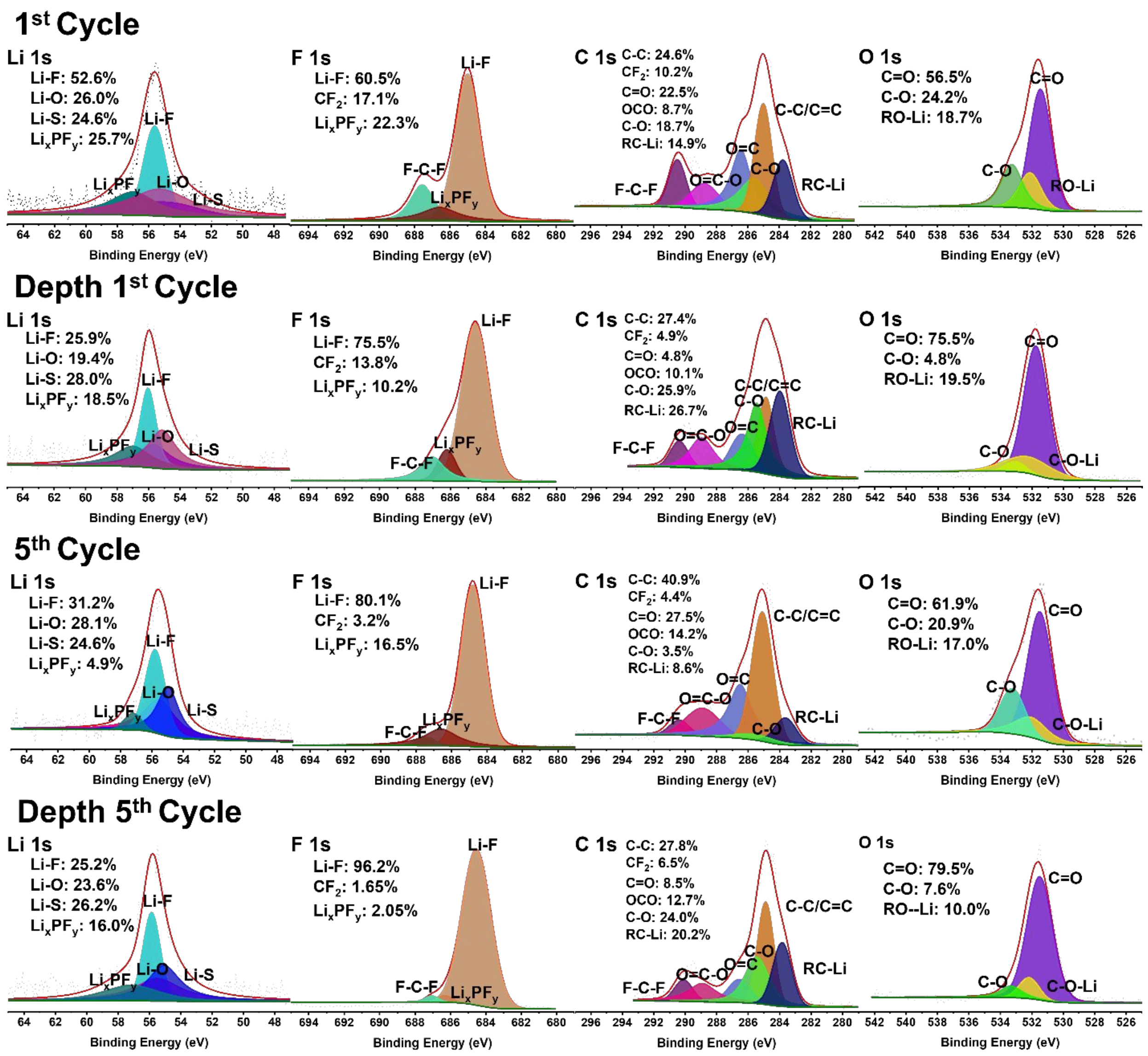

Figure S11. Li 1s, F 1s, C1s, and O1s XPS surface and depth spectra of the Cu_2FeSiS_4 anode at 1st cycle and 5th cycles. Time for initial surface spectra time is 0 and depth spectra is 3 minutes.

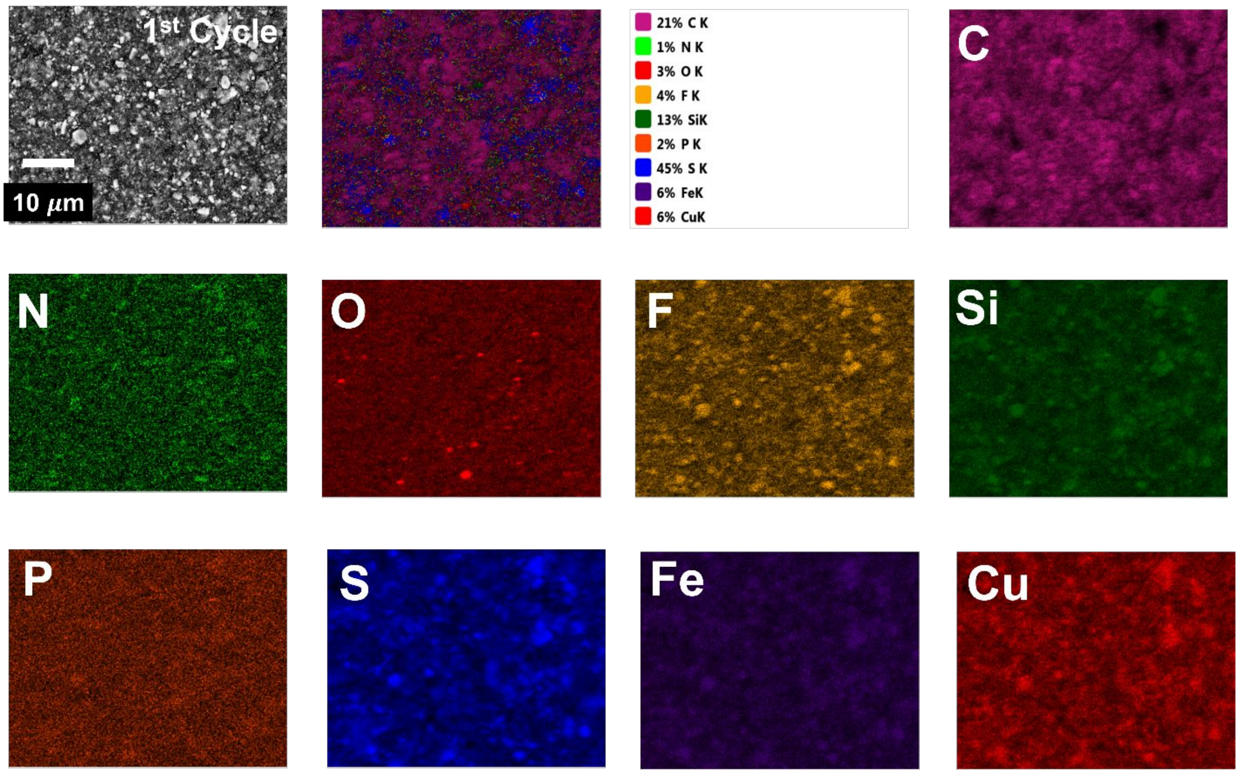

Figure S12. EDS elemental maps of the 1 cycled Cu_2FeSiS_4 anode.

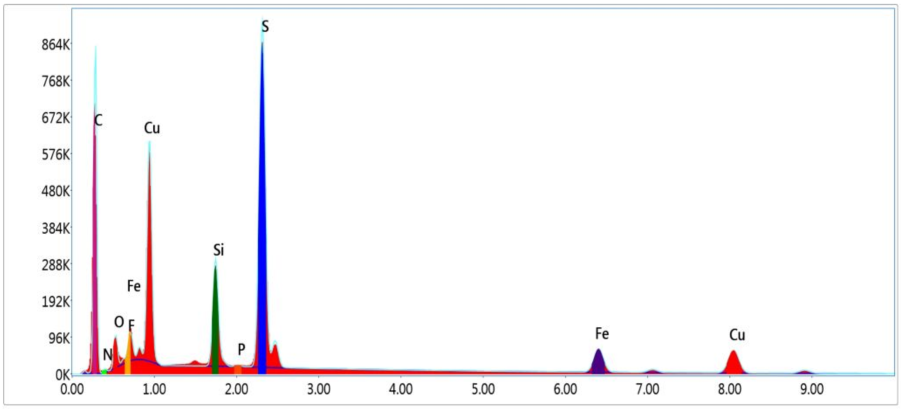

Element	Weight %	Atomic %	Net Int.	Error %	R	A	F
C	54.06	77.86	1040.26	10.43	0.8957	0.0824	1.0000
N	0.96	1.19	12.48	11.91	0.9024	0.0553	1.0000
O	3.34	3.61	120.66	10.22	0.9078	0.1245	1.0000
F	0.43	0.39	32.64	9.84	0.9128	0.2375	1.0000
Si	3.52	2.17	589.29	4.19	0.9339	0.7281	1.0123
P	0.08	0.04	10.03	7.12	0.9375	0.7861	1.0205
S	15.93	8.59	2146.44	3.01	0.9410	0.8470	1.0084
Fe	6.47	2.00	227.81	3.16	0.9720	0.9868	1.1358
Cu	15.20	4.14	247.71	3.39	0.9814	0.9910	1.0657

Figure S13. EDS elemental analysis of the cycled Cu_2FeSiS_4 anode.

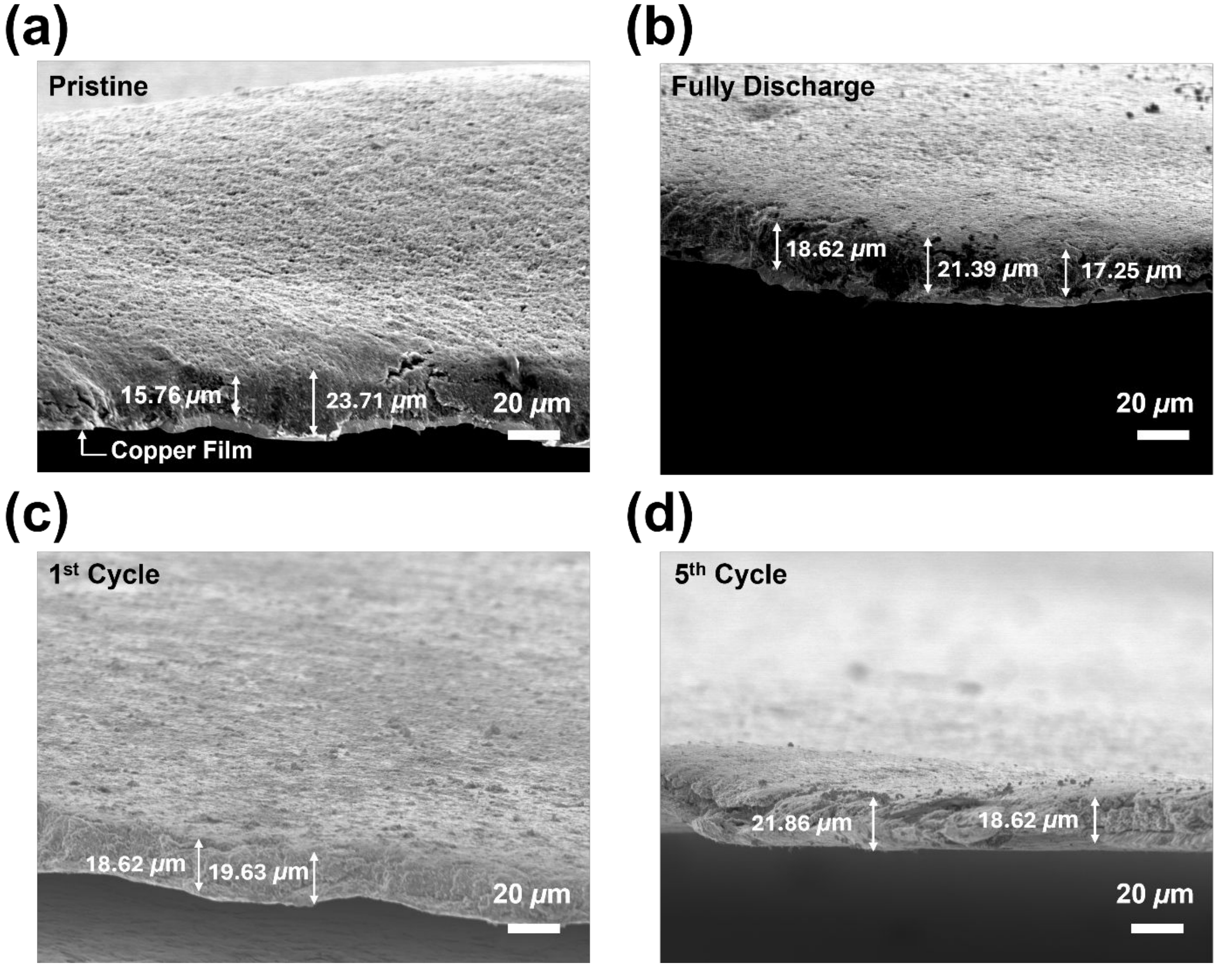

Figure S14. Cross-sectional SEM images. (a) pristine; (b) first fully discharged; (c) 1st cycled; (d) 5th cycled electrode.

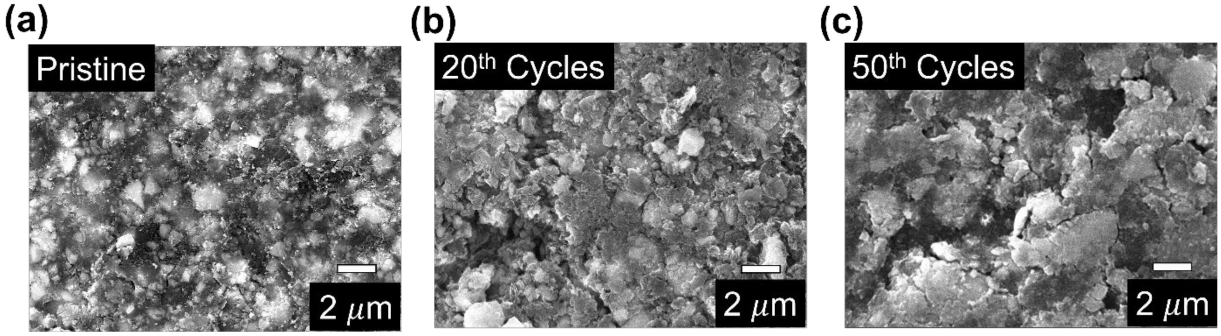

Figure S15. SEM images of the (a) pristine and (b,c) cycled Cu_2MnSiS_4 anodes.

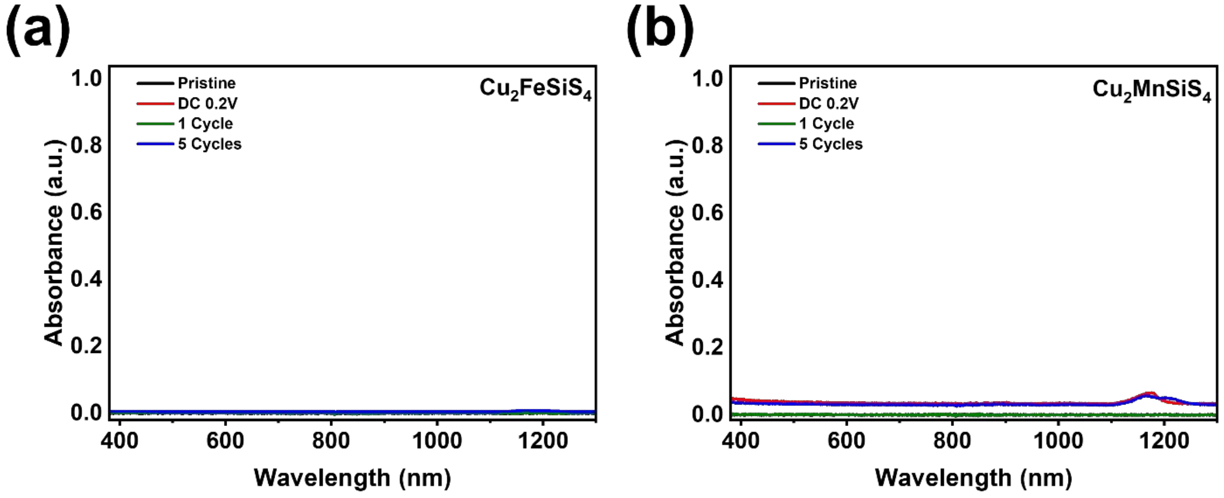

Figure S16. Solubility test for composite electrodes in the electrolyte, 1M $LiPF_6$ in EC:DEC (1:1/V) with FEC (10%). UV-vis spectra of electrolyte. (a) Cu_2FeSiS_4; (b) Cu_2MnSiS_4.

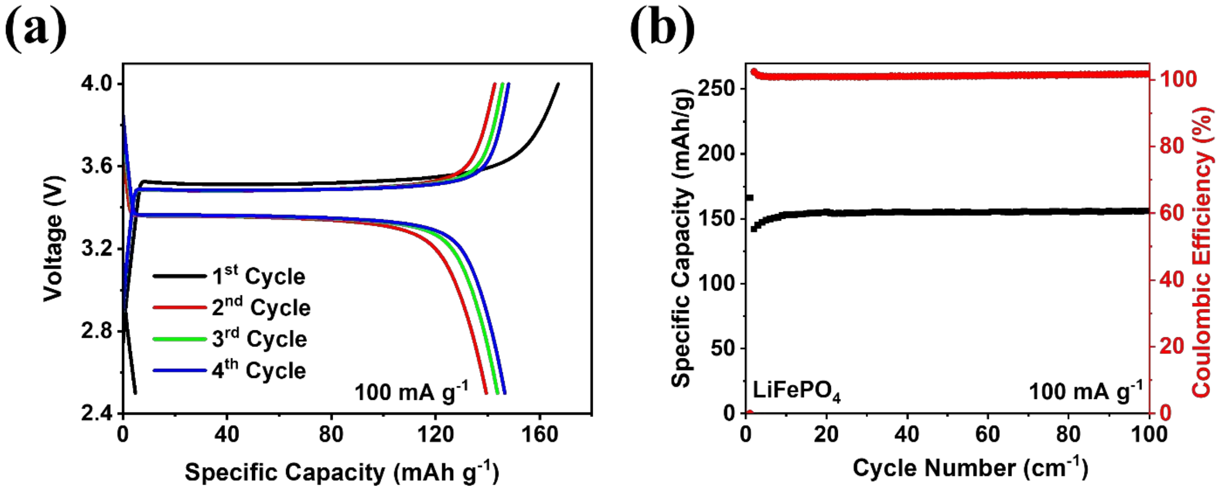

Figure S17. (a) Galvanostatic charge-discharge curves of $LiFePO_4$ in 1M $LiPF_6$ in EC:DEC (1:1 /V) with 10 % FEC; (b) Cycle life and Coulombic efficiency of $LiFePO_4$ half-cell upon long-term cycling.
